# 

**Published:** 1905-05

**Authors:** 


### CONTENTS.

ORIGINAL COMMUNICATIONS—

ECLAMPSIA. By Frances Bradley, M.D. .... 73
THE ALBUMINURIA OF PREGNANCY. By H. McHatton, M.D. .... 79
SOME UNUSUAL CASES OF INTESTINAL DISEASES. By Henry Slack, Ph.M., M.D. .... 83
PROSTATIC MASSAGE. By W. L. Champion, M.D., Atlanta, Ga .... 89
PROFESSIONAL CONSCIENCE. By Richard Douglas, M.D., Nashville, Tenn .... 93
A LAYMAN'S VIEW OF TUBERCULOSIS. By Eli P. Smith .... 102

EDITORIAL NOTES AND COMMENTS—

THE ATLANTA MEETING OF THE MEDICAL ASSOCIATION OF GEORGIA .... 117
CONVENTION OF AMERICAN ANTI-TUBERCULOSIS LEAGUE .... 121
THE COMMENCEMENT EXERCISES OF THE ATLANTA COLLEGE OF PHYSICIANS AND SURGEONS .... 123
SECRETARY'S REPORT GEORGIA STATE COMMISSION ON TUBERCULOSIS .... 126

CONTENTS CONTINUED .... x

